# What Faculty Want: Academic and Community Emergency Physicians’ Perceptions of Learner Feedback

**DOI:** 10.7759/cureus.23546

**Published:** 2022-03-27

**Authors:** Yusuf Yilmaz, Kay Wu, Parnian Pardis, Rana Kamhawy, Shawn Mondoux, Teresa M Chan

**Affiliations:** 1 Continuing Professional Development, McMaster University, Hamilton, CAN; 2 Department of Medical Education, Ege University, Izmir, TUR; 3 School of Medicine, McMaster University, Hamilton, CAN; 4 Emergency Medicine, McMaster University, Hamilton, CAN

**Keywords:** academic emergency physicians, community emergency physicians, mixed methods, student evaluation of teachers, faculty development

## Abstract

Introduction

Faculty development is often deployed by central medical schools, with little guidance from end-users. How and what faculty members can use to improve their performance requires a deeper understanding from this user group. This study aims to explore how faculty perceive learners’ feedback about their performance as educators.

Methods

This study is an explanatory mixed-method research, wherein community- and academic-based emergency medicine faculty members from nine regional hospitals were surveyed about their perceptions of various outcome measures for faculty development. Selected participants were invited to follow-up interviews. We analyzed the physicians’ perceptions toward teaching and performance feedback data based on faculty’s gender, role as academic or community physician, and work experience.

Results

The quantitative phase has 104 participants, and 15 of these were followed up with interviews. The gender of faculty does not have statistical or practical differences regarding their perceptions of learner feedback. Type of practice contains meaningful insights about the perception of learner feedback although it does not have a statistical difference. Moreover, an inverse trend exists between the physicians’ years of experience and their perceived value of learner feedback. Kruskal-Wallis test showed a significant difference in the faculty’s experience level and their perceived value for the metric “quantity of feedback commentary compared to their peer group” (H(4) = 12.21, p = 0.02), specifically between junior and senior faculty (p = 0.007). Some faculty stated that experienced faculty may perceive they have a very well-established style.

Conclusions

Diversifying feedback sources and delivery may be useful for different groups of faculty members. Junior physicians are more interested in gaining feedback about the quantity of their written feedback to students compared to more senior physicians. Learner feedback holds promise to trigger continuous improvement in community sites for those who fall behind compared to the academic sites.

## Introduction

Feedback is critical to enhance the education and training of clinical faculty members and students alike [1]. Traditionally, student evaluations have been the main source of feedback provided to faculty members. These student evaluations provide a foundation for decisions regarding changes to the curriculum, faculty tenure, and promotions as well as refinement of educational materials and tools developed by the faculty [2,3]. However, increasing evidence suggests that these evaluations may be wrought with bias [4-6].

A short history of faculty feedback

The mainstay of feedback for faculty members is often student evaluations that are fairly limited in their use for faculty development purposes [7]. Several issues with the collection and analysis of feedback for faculty members result in a debate surrounding their overall utility in academia. Some of the issues arise from a lack of institutional culture conducive toward learner feedback [4], the need for specific feedback [5], and triangulating learner’s feedback with other activities of faculty members [6].

Institutions often collect students’ feedback through surveys that include closed-ended choice selections and open-ended free text questions, generating a mixture of quantitative and qualitative feedback [8]. Methods for analyzing data and reporting back to faculty are wide-ranging and infrequent - often a single faculty member will receive a report each year that amalgamates several learner groups into one report containing simple descriptive statistics (e.g., average teaching efficacy), and providing an aggregated list of anonymized students’ feedback to teachers often makes feedback ambiguous and non-specific. Using large swaths of aggregated analytics (e.g., clinical supervision over a six-month or 12-month period) creates ambiguous and overly generalized student responses. The literature suggests that faculty members prefer more specific and often more timely feedback for their teaching activities [4]. Other types of outcomes for measuring faculty performance are starting to emerge, especially in the setting of competency-based medical education [9].

What do faculty members want?

There exists a paucity of evidence around what frontline clinical teachers want as feedback to potentiate their performance. There is little work that articulates the impact of student feedback (or other feedback data) on teaching instruction or the enablers of transformative practice feedback for clinical teachers.

At our institution, our community and academic faculty only receive annual aggregate reports of student or resident evaluations of their teaching once annually. In our effort to generate better performance feedback writ large, we felt it prudent to engage in a dedicated exploration of how faculty members perceive and react to their feedback and to determine which report tools are more suitable for faculty members as the current literature overlooks this analysis.

This study aims to explain how various cohorts of faculty members perceive the feedback they receive about their performance as educators. We hope that gaining a better understanding of the types of teaching performance feedback that our faculty would want may help us to create methods to more effectively understand their present performance and set goals to further develop themselves.

## Materials and methods

Context

McMaster University has affiliated hospitals at three campuses (Hamilton, Niagara, and Kitchener-Waterloo). Emergency medicine physicians practicing within these regional campuses and other hospitals within the catchment area are eligible to become faculty members, which is a result of their teaching responsibilities to medical students rotating through these sites.

Research design

As part of a larger program of scholarship, we developed an explanatory mixed-methods study design [10], which included two phases. First, we engaged a larger group of faculty members in a regional need assessment survey [11]. Subsequently, we conducted a follow-up series of interviews with a select cohort of volunteers from within the surveyed population.

We used this design so that we could gather an overarching understanding of how our faculty felt about the feedback they received but then harness the power of in-depth interviews to gain a deeper understanding of the nuances of how a smaller cohort of individuals perceived the patterns in this survey data.

Quantitative phase

Survey Development

The overall survey included questions regarding our participants’ demographics and their attitudes about the feedback that they receive about their teaching and/or supervisory practices. All questions were evaluated on a 10-point Likert scale. The survey was piloted with two non-participatory faculty members (who are experts in health research methods) for content validity and flow. Based on their feedback, the survey was refined and then converted to an online form in SurveyMonkey® (Momentive, California, USA) [12]. Our senior author (TC) then reviewed the survey once more for content, validity, and language refinement. The final version of the survey was reviewed for readability, consistency, comprehension with face, and content validity in mind by other researchers who took part in the parent project [11].

Sampling and Data Collection

We distributed our survey link by emailing the emergency department (ED) chiefs at various departments within our McMaster-affiliated teaching hospitals (nine local health systems). The clinical chiefs shared the link with their faculty via email. This is the overall quality improvement survey that also included items about clinical teaching performance (i.e., teaching quality as well as generating adequate feedback and assessments for trainees).

This study reports the part of the survey regarding performance metrics about teaching and supervisory performance. The recruitment email was initially sent in January of 2019, and then follow-up reminders were sent in February and March of that same year. The target population for the total number of emergency medicine physicians was 195, and we aimed to reach at least half of the population.

Survey Items and Data Analysis

Our research focused on the demographics of the participants for gender, type of practice, and years of experience in the practice as independent variables. We compared these variables with four dependent variables in terms of perceived importance of feedback as faculty: (1) feedback from medical students, (2) feedback from residents, (3) rate of assessment form completion for learners, and (4) quantity of commentary faculty member generates compared to their peers (e.g., number of workplace-based assessments completed and number of words generated as evidence participation in feedback to trainees). Previous work by our group has found that high-quality comments require higher word counts [13], and hence, we have parlayed that finding into this present study. This metric has also recently been defined by our national society as a metric associated with the quality of education [9].

Means and standard deviations were calculated for each answer within the survey. Using a Mann-Whitney test, we compared differences between the faculty members at our academic sites within Hamilton (i.e., academic physicians) and those within the regional/community hospitals (i.e., community physicians). We also compared our participants’ answers based on their gender using the Mann-Whitney test. To detect any differences between the groups based on their years of experience, we conducted the Kruskal-Wallis test. A Bonferroni correction was applied to set our significance value (p-value) to ensure that we adjusted for pairwise comparisons. Since we compared five different cohorts, our adjusted p-value threshold was determined to be 0.01 [14]. Test-by-test exclusion technique was applied for the missing data. IBM SPSS Statistics for Windows version 26.0 (IBM Corp., Armonk, NY) was used for the analysis. Missing data were excluded pairwise.

Qualitative phase

Sampling

Using intentional sampling, we recruited 15 participants to follow up the quantitative phase. Out of the volunteering participants, intentional sampling was used to represent a diverse sampling of our population (e.g., various genders, different practice locations, varying years of experience, or academic rank). Upon analysis, we found that our thematic sufficiency seemed to occur around 12 transcripts, so no additional participants apart from 15 participants were interviewed.

Interview Procedure

Our team member (SM) developed a semi-structured interview guide based on the survey to explain some of the questions in-depth. Our senior author (TC), who has qualitative research experience, reviewed this initial draft of the interview guide and was used as a pilot participant to train our interviewer (RK), and her feedback was used to further refine the interview guide. The final interview guide is found in Appendix A.

Data Collection

One of our team members (RK) conducted the interview from July to September 2019. The audio from the interviews was recorded, and the written documents were generated by transcribing the interviews. A trained medical transcriptionist redacted the transcripts, providing an alias for each participant.

Data Analysis

We used a generic qualitative method to code and interpret the themes within the transcripts. For the qualitative analysis, we involved four team members with varying perspectives to ensure a robust analytic process. One member (YY), who is a PhD-trained education scientist and is a non-clinician providing an outside perspective on the data, was the team lead. Two members are medical students (PP and KW) who have been at various teaching sites within the diverse McMaster campus and provided a trainee’s point of view on the data. The fourth member (TC) is our senior author who is a clinician-educator and faculty developer within our community who brought her experience and content expertise.

We used a simple group coding technique to analyze qualitative data. Four of our team members (YY, PP, KW, and TC) met to create a coding schema based on the first transcription [15]. Subsequently, the team met an additional four times, each time iteratively adding new codes and organizing the data. An audit trail was generated, and two other members of the team (SM and RK) audited the analysis to ensure trustworthiness.

Ethics

Our study was granted an exemption from the Hamilton Integrated Research Ethics Board, as it was deemed a quality improvement project.

## Results

Demographics

A total of 104 emergency medicine physicians (52% response rate) filled out the survey. Of the participants, 63% were male, 61% had primary practice as academics, and a significant number of the participants (40%) had been in practice for less than five years. Seventeen participants had more than 20 years of experience (16%). Only some of the interview participants (n = 10) felt comfortable enough to fill out the demographics section of the survey; the rest preferred to remain anonymous. We reported their demographics in Table 1, but we excluded them in the group comparisons. Other missing data were excluded from the analysis with the test-by-test technique. We also merged mixed practice types of physicians with academic practice types as their duties involve academic tasks in quantitative analysis.

**Table 1:** Demographics of participants by phase

	Quantitative Phase (n = 104)		Qualitative Phase (n = 15)	
Demographic Feature	n	%	n	%
Gender				
Male	65	63	9	60
Female	38	37	6	40
Other	1	1	-	-
Primary Practice Type				
Academic	63	61	7	47
Community	35	34	6	40
Mixed	6	6	2	13
Years of Experience				
0-5	42	40	8	53
6-10	23	22	1	7
11-15	12	12	1	7
16-20	10	10	5	33
20+	17	16	-	-

We interviewed 15 participants recruited from the quantitative phase, in which nine (60%) were male participants. While many participants worked in the academic setting (n = 7), two participants had mixed practice types. The interviews lasted between 33 and 70 minutes.

Gender differences

There were no significant differences between those faculty identifying as male and female in terms of the importance placed upon feedback from medical students (Mdn = 6.00, Mdn = 7.00, U = 892.50, z = -1.109, p = .268) and residents (Mdn = 7.00, Mdn = 7.00, U = 1,027.00, z = -.044, p = .965), rate of evaluation completion (Mdn = 5.00, Mdn = 6.00, U = 1,004.00, z = -.225, p = .822), and quantity of commentary (i.e., word count of feedback written to learners in their assessment forms) compared to their peer group (Mdn = 5.00, Mdn = 6.00, U = 962.50, z = -.090, p = .928), respectively (Table 2).

**Table 2:** Mann-Whitney results by gender U: Mann-Whitney test statistic; z: z-score; p: significance; n: number of participants for the corresponding variable.

	Male		Female		U	z	p
	Median	n	Median	n			
Medical student preceptor evaluation	6	59	7	35	892.5	-1.11	.27
Resident preceptor evaluation	7	59	7	35	1,027	-0.04	.97
Rate of evaluation completion for learners	5	59	6	35	1,004	-0.23	.82
Quantity of commentary compared to their peer group	5	59	6	33	962.5	-0.09	.93

Our qualitative analysis did not reveal any differences in results between male and female physicians. Physicians identifying as both male and female physicians noted similar perspectives on the feedback that they receive. One junior physician, “You want more feedback about how you are doing both from ... your peers and from the trainees,” but “after you have been practicing for a long time ... you probably have a pretty set practice pattern and are less interested in improving.” Furthermore, all physicians similarly attributed the culture of the learning environment to impact the value physicians place upon feedback and feedback delivery. Participants explained their perception of the feedback as that “people who have trained more recently know the value of good feedback when you are learning and trying to figure out how to be a physician” and that “people who are earlier in practice may be more aware of the current construction of our new types of assessments (and) feedback systems.”

Type of practice

There were no significant differences between the academic physicians and the community physicians in terms of the importance placed upon feedback from medical students (Mdn = 7.00, Mdn = 5.00, U = 741.00, z = -1.670, p = .095) and residents (Mdn = 7.00, Mdn = 7.00, U = 925.00, z = -.145, p = .885), rate of evaluation completion (Mdn = 6.00, Mdn = 5.00, U = 848.50, z = -.777, p = .437), and quantity of commentary about learners written by faculty within assessment forms compared to their peer group (Mdn = 6.00, Mdn = 5.00, U = 852.00, z = -.522, p = .602), respectively (Table 3).

**Table 3:** Mann-Whitney results by the type of practice U: Mann-Whitney test statistic; z: z-score; p: significance; n: number of participants for the corresponding variable.

	Academic physicians		Community physicians		U	z	p
	Median	n	Median	n			
Medical student preceptor evaluation	7	65	5	29	741	-1.67	.10
Resident preceptor evaluation	7	65	7	29	925	-.15	.89
Rate of evaluation completion for learners	6	65	5	29	848.50	-.78	.44
Quantity of commentary compared to their peer group	6	63	5	29	852	-.52	.60

Qualitatively, participants revealed several differences in the academic and community physicians’ motivations for and experience with the feedback. Academic centers tend to place a greater emphasis on continuous improvement due to physicians’ teaching role and the continued presence of learners. One participant elaborated, “In an academic setting you are ... geared to continuous education, continuous learning, continuous improvement. You are teaching, you have learners. So, you have to set an example for those learners, and you have to be able to teach them and prepare them for their careers. So, you want to improve yourself first before you can do all of that.” This is reflected in the number of evaluations conducted in academic settings.

In addition, the feedback process may bring more value to academic physicians as it may impact career goals, "The expectation is that it is (needed) in order to get a teaching position or to get higher up in the teaching process. It is expected of you to be part of the teaching and academic community and to be under scrutiny ... because students evaluate you and what kind of teacher you are." This presence of a well-established feedback culture in academic centers compared to the community was highlighted by several participants. One community physician stated "(The academic setting) is ... a very evaluation-driven type of environment just because that is the expectation when you are teaching. ... There is evaluation everywhere, for every student, every learner, every month. ... Maybe it normalizes things for them a little bit."

In contrast, community physicians’ motivations for valuing feedback may differ due to the potentially greater number of opportunities they have for improvement. One community physician stated that "Community doctors recognize that ... when you are practicing in a community and not an academic environment, ... you do start to fall behind. ... I think community physicians recognize that there are always going to be areas in their practice that (they) have a broad ability to improve. But (because) academic physicians are for the most part ... up to date, ... they probably don't feel that there (are) quite as many opportunities (to improve)." Thus, community physicians may value feedback for different reasons compared to academic physicians, which is supported by the lack of difference in quantitative rating of importance between the two groups.

Years of experience

There were no significant differences in the years of experience in practice in terms of the importance placed upon the feedback from medical students (p > .088) and residents (p > .051), and rate of evaluation completion (p > .191). However, the quantity of feedback commentary about learners written by faculty in the assessment forms compared to their peer group was significantly less valued by those with more experience in practice (H(4) = 12.21, p = .016). Bonferroni correction with an alpha level of .01 was used for posthoc pairwise comparison tests. There was a significant difference only between junior faculty (<5 years) (Mdn = 7.00) and senior faculty (>20 years) (Mdn = 3.50, p = .007, r = .47). None of the other comparisons were significant after the Bonferroni correction (p > .012). Table 4 shows the details of the test statistics.

**Table 4:** Kruskal-Wallis results by years in practice H: Kruskal-Wallis test statistic; df: degrees of freedom; p: significance; n: number of participants for the corresponding variable. *Significant p-value.

Evaluation Type	Years in Practice	Median	n	H	df	p
Medical student preceptor evaluation	<5 years	7	38	8.10	4	.09
	5-10 years	6	20			
	11-15 years	6	10			
	16-20 years	7	10			
	>20 years	5	16			
Resident preceptor evaluation	<5 years	8	38	9.46	4	.05
	5-10 years	6.5	20			
	11-15 years	7.5	10			
	16-20 years	7	10			
	>20 years	5.5	16			
Rate of evaluation completion for learners	<5 years	7	38	6.12	4	.19
	5-10 years	5	20			
	11-15 years	5	10			
	16-20 years	6	10			
	>20 years	4.5	16			
Quantity of commentary compared to their peer group	<5 years	7	38	12.22	4	.02*
	5-10 years	6.5	20			
	11-15 years	5	10			
	16-20 years	5	10			
	>20 years	3.5	14			

The qualitative analysis revealed insights about how participants who have various years of experience perceived feedback. Junior physicians may be keener to learn. One participant explained, “... When you are more junior, you are just honing your teaching skills. And you probably want more feedback because it is the only kind of feedback that you have about that.” Similarly, others stated that “They still know that there are lots to learn,” as “earlier on you are still trying to figure out … what your teaching style is,” and consequently “you may need more validation at that time.”

Furthermore, junior physicians may have been trained in a feedback-heavy culture, making them more amenable to feedback. One participant stated that “People who have trained more recently know the value of good feedback.” The quantitative findings may be reflective of differences in previous experience: senior physicians may simply “... find completing the evaluations ... less helpful because they are more used to doing ... verbal ... feedback.” As well, “... in some of the academic centers, there (are) a lot of regular daily evaluations, and people may feel a little bit inundated with the evaluation process.”

On the other hand, as physicians progress through their careers and become more experienced, some may not place as much value on feedback, while others may continue to strongly value feedback. The qualitative analysis uncovered many potential reasons for the former. With more experience, many participants indicated that some physicians may reach a comfort zone. These physicians are comfortable with their practice and subsequent outcomes, making them less amenable to change. Thus, they might think that feedback is a waste of their time, "People (with more) than 20 years (of experience) may have a very well-established teaching style. They may have already figured out what works and what doesn't work for them and for a lot of learners with the experience that they have. So, maybe that type of data is less useful for them.” Furthermore, “sometimes it is even harder to unlearn what you've learned."

Other highlighted reasons included changing career priorities and burnout. One participant stated, "There are other things in your career that are starting to take precedence, (such as) research or anything in the academic stream. ... If you look at ... the average ... age for individuals, that might also correspond to ... family commitments ... starting to become more relevant and ... important ... than ... your work environment." As well, community physicians with many more years of experience may simply be disconnected from the academic, feedback-heavy environment: "You see less value because you are further from your own training that you see that you have forgotten the value that that feedback can have on your own clinical practice. And you have also in our current model spent 20 years not getting feedback. So how are you going to be any good at giving feedback if you haven't gotten it for 20 years?"

However, some physicians with more years of experience may continue to strongly value feedback due to a desire to learn intentionally to stay up-to-date and to engage in mentorship. As a more experienced participant indicated, “Us folks that are getting ... closer to 20 years ... are so far out of the academic zone at that ... we have to take intentionality around our learning to kind of get ourselves up to date on topics.” Another participant pointed out that “The training environment that we have right now changed because of the senior physicians who have been working for 20 years. So, they brought the change.” This is supported by the quantitative lack of difference in the importance placed upon feedback and rate of evaluation completion observed.

## Discussion

In this study, we examined the faculty members’ perceptions of the feedback that they have received about their performance as teachers. We found that there was an inverse trend with increasing experience, faculty members valued student and trainee feedback less, but the difference was not statistically significant. However, senior faculty seemed to then prefer peer-comparison data for hard metrics such as word counts in their comments in workplace assessments about trainees. This finding was not surprising to us but warrants some further exploration in future work. We hope this may have to do with their experience with student comments being unreliable or easily affected by external factors (e.g., cookies) [2]. Junior faculty, on the other hand, highly value student trainee feedback as they feel it helps them in developing their roles as a teacher. Interestingly, there were few differences between the faculty of different genders and also little difference between our community-based and main-campus physician teachers with regard to their preferences for faculty performance feedback - all groups valued feedback about their faculty performance equally.

Our present study must be contextualized within the broader field of feedback within faculty development. Institutions often collect students’ feedback through surveys that include closed-ended choice selections and open-ended free text questions, generating a mixture of quantitative and qualitative feedback, respectively [8]. The feedback faculty do receive is often cumulative for the department or division rather than targeted to their specific performance. Methods for analyzing data and reporting back to faculty members are wide-ranging and infrequent. Using simple descriptive analytics (e.g., average teaching efficacy) and providing an aggregated list of anonymized students’ feedback to teachers often make feedback ambiguous and non-specific. Using large swaths of aggregated analytics (e.g., clinical supervision over a six-month or 12-month period) creates ambiguous and overly generalized student responses. Faculty members prefer more specific and often more timely feedback for their teaching activities [4].

Faculty teaching performance data, therefore, is an area that our participants deemed worthy of continued exploration. However, there is controversy around the nature and kinds of data that might drive performance change. Junior faculty seemed to value trainee feedback more, but we feel that this may be because it is a limited pool of data that they receive about this part of their new identity as teachers. For those just beginning their careers, learner feedback was seen as direct input into their development as new faculty. Supporting our junior colleagues with feedback to help them develop as clinical teachers is certainly seen as valuable by the participants in our study and mirrored in the literature [16-18].

However, it is also unsurprising that the more senior faculty in our study thought very little of student or trainee evaluations of their performance. The literature is filled with examples of how learner evaluations of faculty are fraught with problems. Student evaluations of teachers (SET) tend to be biased in several ways - studies have shown higher ratings for male faculty [16,19,20], those who are considered more attractive [21-23], and those who feed them cookies [2]. Some studies have found that SETs may not even measure effectiveness in teaching [24]. As such, it is no wonder that more senior faculty preferred more quantitative measures (e.g., average word counts for assessments they completed on trainees, as compared to their peers, or several assessments completed).

Interestingly, when asked about faculty feedback, many of our participants found it hard to disentangle their own clinical performance feedback from their faculty performance feedback. Previous studies have found that clinical faculty members in emergency medicine often see themselves as holding three different unique roles (teacher, assessor, and patient protector) when supervising trainees in the clinical environment [25]. This perception, therefore, may affect the way they see themselves in terms of the type of faculty feedback performance analytics they would prefer; to be good teachers, they must get feedback about trainee experience, whereas as assessors, they likely need feedback about the assessment data they generate but also they wish to have insights into trainee and peer perceptions of their clinical care and abilities to function as patient protectors or direct healthcare providers.

Next steps

The next step, therefore, is to operationalize and create reporting mechanisms that can provide faculty with the feedback they both need and want. Our participants have indicated that there is an appetite for possessing data about one’s faculty performance. Knowing the preferences of our faculty members should help to design a better reporting system that bears in mind the perspectives of our various subgroups of faculty.

Likely, to suit the needs of both junior and senior faculty, we need to present them with data that speaks to their unique needs. Junior faculty will want reassurances and comments that provide them with a better understanding of their learners’ experiences - they will want to know that they are creating positive learning environments and being responsive to learners’ needs as it is important for their early career identity formation as teachers. Meanwhile, senior faculty want objective data that allows them to see how they are performing compared to their peers.

To date, this has not been the norm; in the age of competency-based medical education (CBME), there is an opportunity for us to generate more objective data for senior faculty members. In 2018, the Royal College of Physicians and Surgeons of Canada (RCPSC) began implementing the Competence by Design system, which obligates faculty members to generate workplace-based assessments to attest to trainees’ achievement of specific tasks (or entrustable professional activities). This type of performance data can be seen as a great opportunity to gain access to new quantitative metrics for generating faculty performance feedback: Each time a faculty writes a comment about a resident, we can gather data on their contributions as a faculty member to that trainee. Over time, harnessing CBME databases and flipping the data to be about the faculty rather than the trainee will be a valuable source of information to provide objective performance data to our data-hungry senior faculty. Other opportunities for faculty performance might include metrics on other contributions such as scholarly publications, citations, teaching activities, mentorship, and coaching. To provide this type of performance data, what we must first do is find easy-to-achieve methods to log such data and display it for faculty to interpret and make use of.

Limitations

Our data is limited by the extent to which we initially surveyed our participants about their preferences for faculty performance data. Originally, we surveyed our participant pool as part of a larger study and embedded only a small set of questions about clinical faculty performance. Luckily, our qualitative follow-up interviews were able to explain much more about our faculty’s preferences; however, in this group, we lacked insights from more experienced faculty (>20 years). These may limit the generalizability and transferability of our findings, which come from one geographic area.

## Conclusions

While faculty members do value learner feedback to various degrees, different cohorts of faculty may wish to receive different types of feedback. Learner feedback may inform junior faculty development practice; senior faculty members may value more objective data about their performance. Further investigation regarding faculty performance data using an end-user-focused methodology may assist in furthering our understanding of divergent needs within faculty groups.

## Appendices

Appendix A: Questions from the interview guide

A practitioner’s time in practice is almost directly associated with a decrease in their estimation of the value of teaching feedback from both medical students and residents. Why do you think this is the case?

Do you think this is a direct result of duration in practice or simply a difference in the training environments between respondents that are new to work compared to those in practice > 20 years?

This trend also held for the value placed on the completion of evaluations and the quantity of commentary provided. Why do you think practitioners with >20 years in practice rank these elements so low?
